# 4,4′-Dimeth­oxy-2,2′-[(butane-1,4-diyldi­oxy)bis­(nitrilo­methyl­idyne)]diphenol

**DOI:** 10.1107/S1600536810040511

**Published:** 2010-10-20

**Authors:** Yin-Xia Sun, Xiu-Yan Dong, Hu Zhao

**Affiliations:** a School of Chemical and Biological Engineering, Lanzhou Jiaotong University, Lanzhou 730070, People’s Republic of China

## Abstract

The title Schiff base bis­oxime compound, C_20_H_24_N_2_O_6_, lies across an inversion centre and adopts an *E* configuration with respect to the C=N bond. In the mol­ecule, the oxime group is roughly coplanar with the benzene ring, forming a dihedral angle of 1.77 (2)°. An intra­molecular O—H⋯N hydrogen bond forms a six-membered ring with an *S*(6) motif. Weak inter­molecular C—H⋯O hydrogen bonding is present in the crystal structure.

## Related literature

For applications of Schiff base compounds, see: Dong & Ding (2008 ▶); Dong *et al.* (2007 ▶, 2009*b* ▶); Koizumi *et al.* (2005 ▶); Lu *et al.* (2006 ▶). For the synthesis, see: Dong *et al.* (2009*a* ▶).
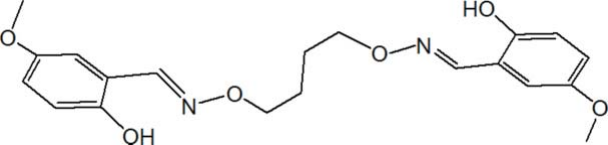


## Experimental

### 

#### Crystal data

C_20_H_24_N_2_O_6_
*M* *_r_* = 388.41
Monoclinic, 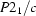

*a* = 4.7310 (4) Å
*b* = 17.1418 (16) Å
*c* = 12.2648 (12) Å
β = 90.981 (1)°
*V* = 994.50 (16) Å^3^
*Z* = 2
Mo *K*α radiation
μ = 0.10 mm^−1^
*T* = 298 K
0.50 × 0.22 × 0.18 mm

#### Data collection

Bruker SMART 1000 CCD area-detector diffractometer
4976 measured reflections
1765 independent reflections
834 reflections with *I* > 2σ(*I*)
*R*_int_ = 0.069

#### Refinement

*R*[*F* ^2^ > 2σ(*F* ^2^)] = 0.050
*wR*(*F* ^2^) = 0.149
*S* = 1.02
1765 reflections
128 parameters
H-atom parameters constrained
Δρ_max_ = 0.17 e Å^−3^
Δρ_min_ = −0.20 e Å^−3^

### 

Data collection: *SMART* (Siemens, 1996 ▶); cell refinement: *SAINT* (Siemens, 1996 ▶); data reduction: *SAINT*; program(s) used to solve structure: *SHELXTL* (Sheldrick, 2008 ▶); program(s) used to refine structure: *SHELXTL*; molecular graphics: *SHELXTL*; software used to prepare material for publication: *SHELXTL*.

## Supplementary Material

Crystal structure: contains datablocks global, I. DOI: 10.1107/S1600536810040511/xu5040sup1.cif

Structure factors: contains datablocks I. DOI: 10.1107/S1600536810040511/xu5040Isup2.hkl

Additional supplementary materials:  crystallographic information; 3D view; checkCIF report

## Figures and Tables

**Table 1:** Hydrogen-bond geometry (Å, °)

*D*—H⋯*A*	*D*—H	H⋯*A*	*D*⋯*A*	*D*—H⋯*A*
O2—H2⋯N1	0.82	1.93	2.643 (2)	145
C3—H3⋯O2^i^	0.93	2.65	3.481 (3)	150
C9—H9⋯O2^i^	0.93	2.51	3.382 (3)	157
C10—H10*A*⋯O3^ii^	0.96	2.74	3.448 (4)	131

Symmetry codes: (i) 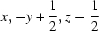; (ii) 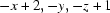.

**Fig. 1.**
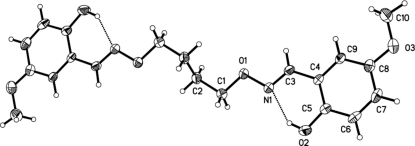
The molecule structure of the title compound with atom numbering [Symmetry codes: -x,-y + 1,-z + 1]. Displacement ellipsoids for non-hydrogen atoms are drawn at the 30% probability level.

**Fig. 2.**
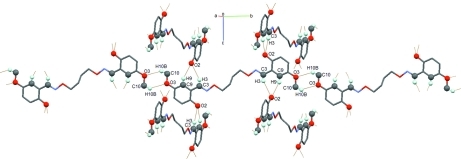
Part of the supramolecular structure of the title compound. Intermolecular hydrogen bonds are shown as dashed lines.

**Fig. 3.**
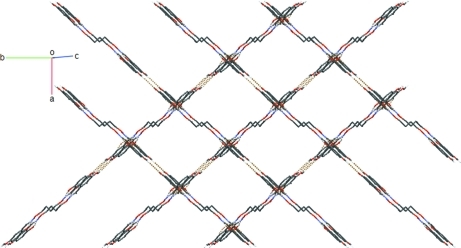
A view of the three-dimensional network for the title compound, and the hydrogen atoms are omitted for clarity.

## supplementary crystallographic information

### Comment

Schiff base compounds are still one of the most prevalent mixed-donor ligands in coordination chemistry (Dong *et al.*, 2007; Dong *et al.*, 2008). In the past decades, a continuing attention has been drawn to the Schiff bases derived from benzaldehyde or salicylaldehyde and their metal complexes for the investigation of single molecule based magnetism, materials science, catalysis of many reactions which could be finely tuned by different substituent groups bonded to the phenolic ring (Koizumi *et al.*, 2005; Dong *et al.*, 2009*b*; Lu *et al.*, 2006). Here, the structural characterization of the title compound, 4,4'-dimethoxy-2,2'- [(butane-1,4-diyldioxy)bis(nitrilomethylidyne)]diphenol, is reported.

The crystal structure of the title compound is built up by only the C_20_H_24_N_2_O_6_ molecules, in which all bond lengths are in normal ranges. The molecule, Fig. 1, lies across a crystallographic inversion centre (symmetry code: -*x*, -*y*, -*z*) and adopts an E configuration with respect to the C=N bond. The asymmetric unit of the compound is composed of one-half of the molecule. The oxime group is nearly coplanar with the benzene ring, making a dihedral angle of 1.77 (2)°. Within the molecule, the planar units are parallel but extend in opposite directions from the methylene bridge. The two benzene rings are parallel to each other with the dihedral angle of 0.00 (0)° and the distance of 1.664 (3) Å. An intramolecular O—H···N hydrogen bond forms a six-membered ring, producing an S(6) ring motif. In the crystal structure, each molecule links six other molecules into an infinite three-dimensional network supramolecular structure (Fig. 2 and 3), which is built from one-dimensional chains *via* three pairs of weak intermolecular C—H···O hydrogen bonds.

### Experimental

The title compound was synthesized according to the references reported by Dong *et al.* (2009*a*). To an ethanol solution (8 ml) of 5-methoxy-2-hydroxybenzaldehyde (304.3 mg, 2 mmol) was added an ethanol solution (4 ml) of 1,4-bis(aminooxy)butane (120.2 mg, 1 mmol). The reaction mixture solution was stirred at 328 K for 4 h. After cooling to room temperature, the formed precipitate was filtered and washed successively with ethanol and ethanol-hexane (1:4), respectively. The product was dried under vacuum to yield 223.3 mg white microcrystal of the title compound. Yield, 52.6%. m.p. 398–399 K. Single crystals were obtained by slow evaporation from a solution of ethanol/dichloromethane (2:1) of the title compound at room temperature for several weeks. Anal. Calcd. for C_20_H_24_N_2_O_6_ (%): C, 61.84; H, 6.23; N, 7.21; O, 24.71. Found: C, 61.87; H, 6.25; N, 7.15; O, 24.75.

### Refinement

H atoms were treated as riding atoms with distances C—H = 0.97 (CH_2_), 0.96 (CH_3_), 0.93 Å (aromatic) and O—H = 0.82 Å. U_iso_(H) = 1.2U_eq_(C) and 1.5U_eq_(O).

### Crystal data

**Table:**

C_20_H_24_N_2_O_6_	*F*(000) = 412
*M**_r_* = 388.41	*D*_x_ = 1.297 Mg m^−^^3^
Monoclinic, *P*2_1_/*c*	Mo *K*α radiation, λ = 0.71073 Å
Hall symbol: -P 2ybc	Cell parameters from 796 reflections
*a* = 4.7310 (4) Å	θ = 2.4–24.5°
*b* = 17.1418 (16) Å	µ = 0.10 mm^−^^1^
*c* = 12.2648 (12) Å	*T* = 298 K
β = 90.981 (1)°	Needle, pale-yellow
*V* = 994.50 (16) Å^3^	0.50 × 0.22 × 0.18 mm
*Z* = 2	

### Data collection

**Table:**

Bruker SMART 1000 CCD area-detector diffractometer	834 reflections with *I* > 2σ(*I*)
Radiation source: fine-focus sealed tube	*R*_int_ = 0.069
graphite	θ_max_ = 25.0°, θ_min_ = 2.0°
φ and ω scans	*h* = −5→5
4976 measured reflections	*k* = −20→18
1765 independent reflections	*l* = −13→14

### Refinement

**Table:**

Refinement on *F*^2^	Primary atom site location: structure-invariant direct methods
Least-squares matrix: full	Secondary atom site location: difference Fourier map
*R*[*F*^2^ > 2σ(*F*^2^)] = 0.050	Hydrogen site location: inferred from neighbouring sites
*wR*(*F*^2^) = 0.149	H-atom parameters constrained
*S* = 1.02	*w* = 1/[σ^2^(*F*_o_^2^) + (0.0562*P*)^2^] where *P* = (*F*_o_^2^ + 2*F*_c_^2^)/3
1765 reflections	(Δ/σ)_max_ < 0.001
128 parameters	Δρ_max_ = 0.17 e Å^−^^3^
0 restraints	Δρ_min_ = −0.20 e Å^−^^3^

### Special details

**Table:**

Geometry. All e.s.d.'s (except the e.s.d. in the dihedral angle between two l.s. planes) are estimated using the full covariance matrix. The cell e.s.d.'s are taken into account individually in the estimation of e.s.d.'s in distances, angles and torsion angles; correlations between e.s.d.'s in cell parameters are only used when they are defined by crystal symmetry. An approximate (isotropic) treatment of cell e.s.d.'s is used for estimating e.s.d.'s involving l.s. planes.
Refinement. Refinement of *F*^2^ against ALL reflections. The weighted *R*-factor *wR* and goodness of fit *S* are based on *F*^2^, conventional *R*-factors *R* are based on *F*, with *F* set to zero for negative *F*^2^. The threshold expression of *F*^2^ > σ(*F*^2^) is used only for calculating *R*-factors(gt) *etc*. and is not relevant to the choice of reflections for refinement. *R*-factors based on *F*^2^ are statistically about twice as large as those based on *F*, and *R*- factors based on ALL data will be even larger.

### Fractional atomic coordinates and isotropic or equivalent isotropic displacement parameters (Å^2^)

**Table:**

	*x*	*y*	*z*	*U*_iso_*/*U*_eq_	
N1	0.4274 (5)	0.32971 (13)	0.62366 (17)	0.0426 (7)	
O1	0.2476 (4)	0.37363 (10)	0.55503 (13)	0.0495 (6)	
O2	0.6869 (5)	0.27865 (12)	0.80239 (14)	0.0626 (7)	
H2	0.5744	0.3054	0.7672	0.094*	
O3	1.2686 (5)	0.07207 (12)	0.54439 (19)	0.0669 (7)	
C1	0.1003 (7)	0.43013 (16)	0.6187 (2)	0.0494 (9)	
H1A	0.2336	0.4642	0.6562	0.059*	
H1B	−0.0147	0.4043	0.6726	0.059*	
C2	−0.0830 (7)	0.47634 (15)	0.5412 (2)	0.0486 (9)	
H2A	−0.1998	0.5116	0.5829	0.058*	
H2B	−0.2080	0.4408	0.5021	0.058*	
C3	0.5649 (7)	0.27896 (16)	0.5696 (2)	0.0407 (8)	
H3	0.5341	0.2752	0.4947	0.049*	
C4	0.7676 (6)	0.22699 (15)	0.6220 (2)	0.0381 (7)	
C5	0.8222 (7)	0.22862 (17)	0.7338 (2)	0.0453 (8)	
C6	1.0185 (8)	0.17800 (19)	0.7794 (2)	0.0586 (10)	
H6	1.0521	0.1781	0.8544	0.070*	
C7	1.1646 (8)	0.12723 (18)	0.7136 (3)	0.0589 (10)	
H7	1.2999	0.0942	0.7444	0.071*	
C8	1.1118 (7)	0.12492 (17)	0.6016 (2)	0.0500 (9)	
C9	0.9145 (6)	0.17495 (16)	0.5571 (2)	0.0420 (8)	
H9	0.8787	0.1740	0.4823	0.050*	
C10	1.2332 (8)	0.07222 (19)	0.4288 (3)	0.0747 (12)	
H10A	1.0414	0.0589	0.4099	0.112*	
H10B	1.3588	0.0347	0.3975	0.112*	
H10C	1.2757	0.1232	0.4010	0.112*	

### Atomic displacement parameters (Å^2^)

**Table:**

	*U*^11^	*U*^22^	*U*^33^	*U*^12^	*U*^13^	*U*^23^
N1	0.0462 (19)	0.0427 (14)	0.0387 (13)	0.0063 (13)	−0.0049 (12)	0.0047 (12)
O1	0.0589 (16)	0.0474 (12)	0.0419 (11)	0.0167 (12)	−0.0083 (11)	−0.0002 (9)
O2	0.0703 (18)	0.0798 (15)	0.0374 (11)	0.0180 (14)	−0.0085 (11)	−0.0050 (11)
O3	0.0670 (19)	0.0583 (14)	0.0752 (16)	0.0223 (14)	−0.0005 (14)	0.0021 (12)
C1	0.055 (2)	0.0477 (19)	0.0452 (18)	0.0095 (17)	0.0034 (16)	0.0005 (15)
C2	0.049 (2)	0.0423 (18)	0.0547 (19)	0.0086 (16)	0.0037 (16)	0.0033 (13)
C3	0.048 (2)	0.0414 (16)	0.0321 (14)	−0.0018 (17)	−0.0055 (14)	0.0026 (14)
C4	0.040 (2)	0.0387 (16)	0.0355 (15)	0.0004 (16)	−0.0056 (14)	0.0095 (13)
C5	0.050 (2)	0.0496 (19)	0.0362 (16)	0.0034 (17)	−0.0045 (15)	0.0030 (14)
C6	0.065 (3)	0.072 (2)	0.0387 (17)	0.001 (2)	−0.0138 (17)	0.0172 (17)
C7	0.052 (2)	0.061 (2)	0.064 (2)	0.010 (2)	−0.0105 (19)	0.0201 (18)
C8	0.053 (2)	0.0422 (18)	0.0547 (19)	0.0016 (18)	−0.0003 (18)	0.0074 (16)
C9	0.048 (2)	0.0396 (16)	0.0382 (16)	0.0009 (16)	−0.0062 (15)	0.0078 (13)
C10	0.083 (3)	0.068 (2)	0.074 (3)	0.017 (2)	0.018 (2)	−0.0021 (19)

### Geometric parameters (Å, °)

**Table:**

N1—C3	1.278 (3)	C3—H3	0.9300
N1—O1	1.405 (3)	C4—C9	1.390 (4)
O1—C1	1.432 (3)	C4—C5	1.391 (4)
O2—C5	1.368 (3)	C5—C6	1.382 (4)
O2—H2	0.8200	C6—C7	1.381 (4)
O3—C8	1.372 (3)	C6—H6	0.9300
O3—C10	1.425 (3)	C7—C8	1.392 (4)
C1—C2	1.501 (3)	C7—H7	0.9300
C1—H1A	0.9700	C8—C9	1.374 (4)
C1—H1B	0.9700	C9—H9	0.9300
C2—C2^i^	1.524 (5)	C10—H10A	0.9600
C2—H2A	0.9700	C10—H10B	0.9600
C2—H2B	0.9700	C10—H10C	0.9600
C3—C4	1.451 (4)		
			
C3—N1—O1	111.2 (2)	O2—C5—C6	117.6 (3)
N1—O1—C1	109.33 (19)	O2—C5—C4	122.5 (3)
C5—O2—H2	109.5	C6—C5—C4	119.9 (3)
C8—O3—C10	116.9 (2)	C7—C6—C5	119.9 (3)
O1—C1—C2	107.0 (2)	C7—C6—H6	120.0
O1—C1—H1A	110.3	C5—C6—H6	120.0
C2—C1—H1A	110.3	C6—C7—C8	120.8 (3)
O1—C1—H1B	110.3	C6—C7—H7	119.6
C2—C1—H1B	110.3	C8—C7—H7	119.6
H1A—C1—H1B	108.6	O3—C8—C9	125.3 (3)
C1—C2—C2^i^	113.7 (3)	O3—C8—C7	115.7 (3)
C1—C2—H2A	108.8	C9—C8—C7	118.9 (3)
C2^i^—C2—H2A	108.8	C8—C9—C4	121.1 (3)
C1—C2—H2B	108.8	C8—C9—H9	119.5
C2^i^—C2—H2B	108.8	C4—C9—H9	119.5
H2A—C2—H2B	107.7	O3—C10—H10A	109.5
N1—C3—C4	121.8 (3)	O3—C10—H10B	109.5
N1—C3—H3	119.1	H10A—C10—H10B	109.5
C4—C3—H3	119.1	O3—C10—H10C	109.5
C9—C4—C5	119.4 (3)	H10A—C10—H10C	109.5
C9—C4—C3	118.3 (2)	H10B—C10—H10C	109.5
C5—C4—C3	122.3 (3)		
			
C3—N1—O1—C1	−179.4 (2)	C4—C5—C6—C7	1.5 (5)
N1—O1—C1—C2	178.8 (2)	C5—C6—C7—C8	−1.6 (5)
O1—C1—C2—C2^i^	−64.3 (4)	C10—O3—C8—C9	3.4 (5)
O1—N1—C3—C4	179.2 (2)	C10—O3—C8—C7	−175.7 (3)
N1—C3—C4—C9	−178.2 (3)	C6—C7—C8—O3	−179.8 (3)
N1—C3—C4—C5	0.7 (5)	C6—C7—C8—C9	1.0 (5)
C9—C4—C5—O2	179.6 (3)	O3—C8—C9—C4	−179.5 (3)
C3—C4—C5—O2	0.7 (5)	C7—C8—C9—C4	−0.3 (5)
C9—C4—C5—C6	−0.8 (4)	C5—C4—C9—C8	0.3 (4)
C3—C4—C5—C6	−179.7 (3)	C3—C4—C9—C8	179.2 (3)
O2—C5—C6—C7	−178.9 (3)		

Symmetry codes: (i) −*x*, −*y*+1, −*z*+1.

### Hydrogen-bond geometry (Å, °)

**Table:**

*D*—H···*A*	*D*—H	H···*A*	*D*···*A*	*D*—H···*A*
O2—H2···N1	0.82	1.93	2.643 (2)	145
C3—H3···O2^ii^	0.93	2.65	3.481 (3)	150
C9—H9···O2^ii^	0.93	2.51	3.382 (3)	157
C10—H10A···O3^iii^	0.96	2.74	3.448 (4)	131

Symmetry codes: (ii) *x*, −*y*+1/2, *z*−1/2; (iii) −*x*+2, −*y*, −*z*+1.
